# Appearance-Related Partner Preferences and Body Image in a German Sample of Homosexual and Heterosexual Women and Men

**DOI:** 10.1007/s10508-021-02087-5

**Published:** 2021-10-28

**Authors:** Martin Cordes, Silja Vocks, Andrea S. Hartmann

**Affiliations:** Department of Clinical Psychology and Psychotherapy, Osnabrück University, Knollstraße 15, 49069 Osnabrück, Germany

**Keywords:** Appearance-related partner preferences, Gender, Sexual orientation, Body image, Eating pathology

## Abstract

There is evidence that gender as well as sexual orientation can affect body image. In particular, heterosexual women and homosexual men seem to be more vulnerable to a negative body image compared to homosexual women and heterosexual men. One reason for this may be derived from the fact that heterosexual women and homosexual men try to attract male romantic partners: As men place more importance on physical attractiveness than do women, the pressure to fulfill the sociocultural beauty ideal is thus increased. The present online study investigated differences in appearance-related partner preferences and their associations with measures of body image and eating pathology in homosexual and heterosexual women and men. The non-representative sample consisted of 893 participants (*n* = 201 lesbian women, *n* = 192 gay men, *n* = 349 heterosexual women, and *n* = 151 heterosexual men), who completed silhouette measures assessing their perception and expectations regarding body fat and muscularity of their own body and the body of a potential romantic partner, as well as questionnaires on drive for thinness, drive for muscularity, and eating pathology. Overall, few differences in appearance-related partner preferences emerged between the four groups. However, compared to heterosexual women, homosexual men appeared to prefer higher muscularity in potential romantic partners, which was also associated with increased drive for thinness and muscularity and increased eating pathology. The present findings indicate that, irrespective of sexual orientation, women and men tend to share similar standards regarding their own and a potential partner’s physical appearance, potentially suggesting an increased hegemony of heteronormative beauty ideals in women and men in general.

## Introduction

Gender as well as sexual orientation seem to affect the risk of experiencing body dissatisfaction (e.g., Calzo et al., 2018) and of developing pathological eating behavior (Dakanalis et al., 2015; Keel & Forney, 2013). While women tend to be more dissatisfied with their body weight and shape (Grabe et al., 2008), men seem to be more concerned about muscularity (Dakanalis et al., 2015). The literature suggests that despite an increasing prevalence of body dissatisfaction in men over the past decades (Fiske et al., 2014; Gray & Ginsberg, 2007), women still tend to be more vulnerable to a negative body image compared to men (e.g., Laus et al., 2015; Matthiasdottir et al., 2012; Peplau et al., 2009).

Besides this general gender gap, there is some evidence that gay men suffer from a poorer body image compared to heterosexual men (e.g., Frederick & Essayli, 2016; Jankowski et al., 2014; Morrison et al., 2004; Peplau et al., 2009; but also see Kane, 2010), especially with respect to increased eating pathology and higher drive for thinness and muscularity (e.g., Yean et al., 2013). The findings are somewhat more mixed regarding sexual orientation and body image in women. While some studies indicated little to no difference in body image between lesbian and heterosexual women (e.g., Henn et al., 2019; Morrison et al., 2004; Peplau et al., 2009), others did reveal differences (e.g., Henrichs-Beck & Szymanski, 2017; Polimeni et al., 2009; Yean et al., 2013). For example, in one of the few studies to control for differences in body mass index (BMI) between lesbian and heterosexual women, Alvy (2013) reported that lesbian women displayed lower overall body dissatisfaction and higher satisfaction with various body parts than did heterosexual women.

A possible explanation for the outlined differences in body image between heterosexual and homosexual women and men may be derived from the tripartite influence model of body image (Thompson et al., 1999). According to this model and its adaptations to homosexual women and men (e.g., Huxley et al., 2015; Tylka, 2011; Tylka & Andorka, 2012), there are at least four sociocultural factors which—mediated/moderated by the internalization of the thin/muscular ideal and the tendency to perform body-related upward comparisons—contribute to a negative body image: pressure from the media, peers, family, and partners to conform to the sociocultural body ideal (Tylka, 2011). The latter factor seems to be especially relevant regarding the aforementioned differing vulnerability to a negative body image, as heterosexual women and gay men try to attract male partners, whereas lesbian women and heterosexual men try to attract female partners (Siever, 1994). Both gay and heterosexual men place more importance on the physical attractiveness of a potential romantic partner than do lesbian and heterosexual women (Bailey et al., 1994; Legenbauer et al., 2009; Murnen et al., 2015), and this seems to be even more pronounced when considering long-term rather than short-term relationships (Lucas et al., 2011; Meltzer et al., 2014). Therefore, it is conceivable that heterosexual women and gay men experience more sexual objectification and more pressure to be attractive than do lesbian women and heterosexual men (Engeln-Maddox et al., 2011; Siever, 1994), which may increase the risk of body dissatisfaction and a negative body image (Legenbauer et al., 2009).

However, studies investigating the differences in partner preferences and their associations with body image are still rare. In line with the above-mentioned assumption, in a study applying silhouette measures to gay and heterosexual men, Fussner and Smith (2015) found that the two groups did not differ in the estimation of their actual and ideal body size, but that gay men displayed greater discrepancies between their actual body and the body they believed they should have in order to attract potential romantic partners. Moreover, this discrepancy was associated with increased eating pathology. Another study, by Legenbauer et al. (2009), which is one of the very few to compare homosexual as well as heterosexual women and men, revealed that gay men had the highest scores for internalization of the slender body ideal (see also Yean et al., 2013). Moreover, only in this group was internalization the strongest predictor for the preference of attractiveness in a potential romantic partner. Furthermore, in their adaption of the tripartite influence model to gay men, Tylka and Andorka (2012) reported that the pressure from romantic partners to be more muscular was associated with increased internalization of the mesomorphic body ideal and with increased muscle-oriented enhancement behaviors. However, in contrast to the aforementioned findings, in a study comparing heterosexual women and gay men regarding their preferred partner ideal, Štěrbová et al. (2018) found no difference between the two groups in their preference for the mesomorphic somatotype.

With respect to homosexual women, the literature suggests that lesbians prefer body ideals for themselves that are more corpulent (i.e., more body fat) compared to heterosexual women (Alvy, 2013; Markey & Markey, 2014). Furthermore, in another study, lesbians rated images of other women with higher BMIs as more attractive than did heterosexual women (Swami & Tovée, 2006), which may indicate that they value higher BMIs not only for themselves but also for their partners. In line with this, an investigation of the tripartite influence model in females found that while for lesbian women, female partner pressure was neither associated with internalization of the thin body ideal nor with restrained eating, in heterosexual women, male partner pressure was positively related to both variables (Huxley et al., 2015).

To sum up, the reported findings underline that gender and sexual orientation indeed seem to have an impact on body dissatisfaction and body image. This may be especially true for heterosexual women and gay men, who appear to be most vulnerable to a negative body image and eating pathology (Frederick & Essayli, 2016; Legenbauer et al., 2009; Peplau et al., 2009). As the literature review revealed, there is also some support for the assumption that the preference for male romantic partners may lead to increased sociocultural pressure to conform either to the thin or muscular ideal, which in turn might heighten the risk for a disturbed body image (e.g., Fussner & Smith, 2015; Huxley et al., 2015). Nevertheless, the very small number of existing studies on partner preferences and body image mainly focused on same-sex comparisons of homosexual and heterosexual women and men, respectively (e.g., Markey & Markey, 2014). Beyond this, the reported studies either investigated differences in body ideals which a potential romantic partner should fulfill (e.g., Štěrbová et al., 2018) or differences in the subjective expectations of one’s own appearance in order to attract a potential romantic partner (e.g., Fussner & Smith, 2015), but not both. However, an investigation of both aspects of partner preferences within one study would allow for same-sex comparisons regarding one’s own body as well as for other-sex comparisons regarding the potential partner’s body. Furthermore, especially in men, the use of silhouette measures to assess the perceptual body image (i.e., the mental representation of one’s own body; Thompson et al., 1999) and/or body ideals has often been criticized, as the vast majority of the available instruments are adopted from research on women’s body image. As such, the instruments only focus on body fat and not on muscularity, thus tremendously limiting their validity for men (see Cafri & Thompson, 2004).

Therefore, the current online study aimed to address these gaps by examining a comprehensive sample of heterosexual and homosexual women and men regarding general differences in body image (i.e., drive for thinness and drive for muscularity) and eating pathology, and with respect to differences in the perceptual body image (i.e., actual vs. ideal body). The study’s main focus, however, was on investigating differences in appearance-related partner preferences between the different sexual orientations and their associations with measures of body image and eating pathology. The perceptual body image as well as the partner preferences—comprising both the body ideal for a potential romantic partner and the expectations regarding one’s own appearance in order to attract a potential romantic partner—were assessed using two newly designed silhouette measures (i.e., for women and men) that differentiate between the body fat and the muscularity dimension.

With respect to the perceptual body image, we hypothesized that gay and heterosexual men would not differ in the silhouettes they chose for their actual body and their body ideal (see Fussner & Smith, 2015). However, we assumed that compared to heterosexual women, lesbians would choose body ideals with more body fat (Alvy, 2013; Markey & Markey, 2014).

With regard to the partner preferences, we hypothesized that gay men would choose male body silhouettes representing a body ideal for a potential romantic partner that were both more muscular and thinner (i.e., less body fat) compared to the corresponding male body silhouettes chosen by heterosexual women. We further assumed that heterosexual men would choose female body silhouettes representing a body ideal for a potential romantic partner with significantly less body fat compared to the corresponding female body silhouettes chosen by lesbian women.

Furthermore, we hypothesized that when choosing the body silhouettes they believed would be expected by a potential romantic partner, gay men would select significantly more muscular and thinner (i.e., less body fat) silhouettes compared to those selected by heterosexual men, and that heterosexual women would select silhouettes with less body fat compared to those selected by lesbian women.

We further tested for differences between the body silhouettes the participants would choose for their own ideal body and for the ideal body of a potential romantic partner. However, this question was only investigated for the two homosexual groups, as it requires a correspondence between one’s own sex and the sexually preferred gender. We hypothesized that gay men would report no difference between their own ideal body and the ideal body of a potential romantic partner, and that lesbian women would choose a body silhouette for their own ideal body that would be more muscular and less corpulent (i.e., less body fat) compared to the body silhouette they would choose for the body ideal of a potential romantic partner.

Finally, from an exploratory perspective, we investigated associations between the variables for partner preferences and measures of body image (i.e., drive for thinness, drive for muscularity) and eating pathology.

## Method

### Participants

For the present study, inclusion criteria were a minimum age of 18 years and sufficient language German skills. Participants were recruited via posts on social media, on LGBTI* websites and related online forums, as well as via press releases, leaflets in university buildings, and university mailing lists. Of the 6,058 persons who clicked on the link for the survey, 1,708 participants began the survey (28%). A total of 80 persons had to be excluded due to missing data on gender. As the present study only analyzed the data of persons who specified their gender as female or male and their sexual orientation as homosexual or heterosexual, a further 374 persons had to be excluded (i.e., due to non-binary gender: 43 persons; due to other sexual orientations: 331 persons). From the resulting 1,254 participants, a total of 361 persons were excluded due to non-complete datasets regarding the items of the silhouette measures (Body Image Matrix of Thinness and Muscularity; BIMTM). Therefore, the final sample size of this non-representative sample amounted to *N* = 893 (15%), comprising *n* = 201 lesbian women, *n* = 192 gay men, *n* = 349 heterosexual women, and *n* = 151 heterosexual men.

## Measures and Materials

### Body Image Matrix of Thinness and Muscularity (BIMTM)

The BIMTM is a new silhouette measure for the assessment of perceptual body image, which exists in two versions: one matrix for female bodies (Steinfeld et al., 2017) and one matrix for male bodies (Taube et al., 2017). Each matrix consists of 64 (8 × 8) photorealistic and colored images of either female or male bodies, which were created using the software DAZ 3D Studio 4.9 Pro (DAZ Productions, Inc., Salt Lake City, UT). The bodies are displayed without the head and wearing grey underwear. Along the horizontal axis of the matrix, the bodies vary in body fat ranging from 1 (*very low body fat*) to 8 (*very high body fat*). Along the vertical axis, the bodies vary in muscularity ranging from 1 (*very low muscularity*) to 8 (*very high muscularity*). That is, each of the 64 body silhouettes is characterized by a unique combination of a body fat and a muscularity score.

For the present study, participants were asked to choose a body silhouette that represents (a) their *actual body*, (b) their *ideal body*, (c) the ideal body of a potential romantic partner (*ideal partner*), and (d) the body they believe would be expected by a potential romantic partner (*expected body*). All items on potential romantic partners referred to a long-term rather than a short-term relationship. Both the female and male versions of the BIMTM have proven to be reliable and valid instruments for the assessment of perceptual body image (Steinfeld et al., 2017; Taube et al., 2017).

### Drive for Muscularity Scale (DMS)

The DMS (McCreary & Sasse, 2000; German version: Waldorf et al., 2014) was used to assess attitudes and behaviors toward striving for a more muscular build. It consists of 15 items rated on a 6-point scale ranging from *always* (1) to *never* (6). Following the recommendation of McCreary et al. (2004), the steroid item (Item 10: “I think about taking anabolic steroids”) was excluded as it can lead to floor effects within non-weight-training samples. As higher scores are intended to indicate higher levels of DM, all items were recoded. In the current study, the overall DMS score was used. The DMS yielded good internal consistencies (Cronbach’s alpha) in a mixed-gender sample (females: α = 0.82; males: α = 0.87; McCreary et al., 2004).

### Eating Disorder Inventory-2 (EDI-2)

The EDI-2 (Garner, 1991; German version: Paul & Thiel, 2005) is a self-report questionnaire for the assessment of diverse aspects of eating disorder (ED) pathology. In the current study, only the subscale Drive for Thinness (DT) was used to quantify the desire to become thinner and the fear of gaining weight, respectively. The subscale comprises seven items that are rated on a 6-point scale ranging from *never* (1) to *always* (6). Internal consistencies (Cronbach’s α) for the DT subscale amounted to α = 0.86 for non-clinical women and α = 0.70 for non-clinical men (Paul & Thiel, 2005).

### Eating Disorder Examination-Questionnaire (EDE-Q)

The EDE-Q (Fairburn & Beglin, 1994; German version: Hilbert & Tuschen-Caffier, 2016) is a self-report instrument used to quantify ED pathology. It consists of four subscales: Restraint (5 items), Eating Concerns (5 items), Weight Concerns (5 items), and Shape Concerns (8 items). The items are rated on a 7-point scale ranging from *no days/not at all* (0) to *every day/markedly* (6). In the present study, the EDE-Q total score was used, which has shown excellent internal consistencies (Cronbach’s *α*) in a German community sample (females: *α* = 0.94; males: *α* = 0.91; Hilbert et al., 2012).

### Procedure

Data for the current study were derived from a broader online survey on the relationship between sexual orientation and body image, which was available online between April 2017 and September 2018. The survey was set up using the online tool EFS survey (Questback GmbH, Cologne, Germany). The study protocol was approved by the local ethics committee. All participants provided informed consent.

Upon entering the landing page, participants were given information on the purpose of the study; the study’s aim was not concealed. After providing informed consent, participants answered questions on anthropometric and sociodemographic characteristics. This was followed by the BIMTMs for the assessment of perceptual body image and partner preferences, which were filtered according to the participant’s gender and sexual orientation. Subsequently, participants were presented with various questionnaires on body image and psychopathology. On average, the processing time of the survey was about 38 min. As reimbursement, participants were able to enter a lottery to win one of a total of ten online vouchers worth 20 €.

### Data Analysis

Differences between the four groups (i.e., lesbian women, gay men, heterosexual women, heterosexual men) in terms of demographic and anthropometric data and on the DMS, the EDI-2 subscale DT, and the EDE-Q were analyzed with a series of six one-way ANOVAs, followed by alpha-adjusted (Bonferroni procedure, significance threshold of *p* = 0.0083, see Aickin & Gensler, 1996) pairwise comparisons. Effect sizes were reported using Hedges’ *g*_*s*_ (Lakens, 2013), with *g* = 0.20 representing small effects, *g* = 0.50 representing medium effects, and *g* = 0.80 representing large effects (Cohen, 1992). Due to the ordinal scale level of the BIMTM measures, differences between the four groups in the body fat and muscularity scores on these measures were analyzed with a series of 16 Mann–Whitney *U* tests. To control for alpha inflation, the Bonferroni procedure was applied, which led to a lowered significance threshold of *p* = 0.0031. Differences within the homosexual group regarding one’s own body ideal and the body ideal of a potential romantic partner were analyzed via four alpha-adjusted (Bonferroni procedure, significance threshold of *p* = 0.0125) Wilcoxon signed-rank tests. Following Field (2017), effect sizes for the Mann–Whitney *U* tests and the Wilcoxon signed-rank tests were quantified using Cohen’s correlation coefficient *r,* with *r* = 0.1 representing small effects, *r* = 0.3 representing medium effects, and *r* = 0.5 representing large effects (Cohen, 1992). Associations between the questionnaire data and the silhouette measures were analyzed using Spearman’s rank correlation coefficient *r*_s._ All statistical analyses were conducted using IBM SPSS Statistics 25.

## Results

### Participants

Sample characteristics and differences between the four subgroups can be found in Table 1. While gay and heterosexual men resembled each other in their reported relationship status (single vs. romantic partnership), lesbian women were more likely to be single than heterosexual women. However, a series of Bonferroni-corrected Mann–Whitney *U* tests showed no differences in the BIMTM variables between partnered and non-partnered participants. Both homosexual and heterosexual men had a higher BMI than heterosexual women (*g* = 0.38; *g* = 0.33). Furthermore, gay men were significantly older than lesbian women (*g* = 0.44). Beyond this, gay and heterosexual men were significantly older than heterosexual women (*g* = 0.68; *g* = 0.52). With respect to the educational level, gay men and lesbian women reported fewer years of education than did heterosexual women (*g* = 0.50; *g* = 0.38) and gay men also reported fewer years of education than did heterosexual men (*g* = 0.41). Due to the significant differences between the four groups in terms of age and educational level, both variables were added to the correlation analysis.

**Table 1:** Percentage (%), means (M), and standard deviations (SD) of subgroup demographic, anthropometric, and body image characteristics

Variables	1. Gay (*n* = 192)		2. Lesbian (*n* = 201)		3. Heterosexual female (*n* = 349)		4. Heterosexual male (*n* = 151)		Group comparisons and effect sizes ANOVAs (Hedges’ *g*)
In a relationship (%)	43.75		34.83		63.61		46.36		*p* < .001^a^
	*M*	*SD*	*M*	*SD*	*M*	*SD*	*M*	*SD*	
Age (years)	30.73	11.51	26.06	9.38	24.85	6.51	28.95	10.24	1 > 2** (*g* = 0.44), 1 > 3** (*g* = 0.68), 3 < 4** (*g* = 0.52)
BMI (kg/m^2^)	25.00	6.21	24.16	5.71	23.02	4.45	24.46	3.94	1 > 3** (*g* = 0.38), 3 < 4 ** (*g* = 0.33)
Education (years)	12.29	1.30	12.44	1.13	12.78	0.67	12.75	0.98	1 < 3** (*g* = 0.50), 1 < 4** (*g* = 0.38), 2 < 3** (*g* = 0.41)
DT^b^	2.78	1.25	2.86	1.39	3.20	1.28	2.23	0.94	1 < 3* (*g* = 0.33), 1 > 4* (*g* = 0.49), 2 > 4** (*g* = 0.52), 3 > 4** (*g* = 0.81)
DMS^c^	2.69	0.91	2.12	0.64	2.14	0.87	2.70	0.87	1 > 2** (*g* = 0.73), 1 > 3** (*g* = 0.62), 2 < 4** (*g* = 0.78), 3 < 4** (*g* = 0.64)
EDE-Q^d^	1.38	1.14	1.51	1.37	1.73	1.31	1.01	0.88	2 > 4* (*g* = 0.42), 3 > 4** (*g* = 0.60)

BMI: Body Mass Index; DT: Eating Disorder Inventory-2, subscale Drive for Thinness; DMS: Drive for Muscularity Scale; EDE-Q: Eating Disorder Examination-Questionnaire

*p < .01; **p < .001 (alpha adjustment led to a lowered significance threshold of *p* = .0083)

^a^Chi-square test

^b^*N* = 823: gay: *n* = 168; lesbian: *n* = 188; heterosexual female *n* = 327; heterosexual male *n* = 140

^c^*N* = 801: gay: *n* = 164; lesbian: *n* = 184; heterosexual female *n* = 317; heterosexual male *n* = 136

^d^*N* = 667: gay: *n* = 128; lesbian: *n* = 150; heterosexual female *n* = 279; heterosexual male *n* = 110

Moreover, heterosexual women had higher scores on the drive for thinness subscale compared to gay men (*g* = 0.33) and heterosexual men (*g* = 0.81), but not compared to lesbian women (*g* = 0.26). No differences in drive for thinness were found between gay men and lesbian women (*g* = 0.06), but both groups had higher scores compared to heterosexual men (*g* = 0.49; *g* = 0.52). With regard to the drive for muscularity, gay and heterosexual men did not differ (*g* = 0.01), but displayed higher scores than lesbian (*g* = 0.73; *g* = 0.78) and heterosexual women (*g* = 0.62; *g* = 0.64). Finally, lesbian and heterosexual women reported higher scores on the EDE-Q compared to heterosexual men (*g* = 0.42; *g* = 0.60), but not compared to gay men (*g* = 0.10; *g* = 0.28).

### Differences in the Body Image Matrix of Thinness and Muscularity Measures

#### Comparison of One’s Actual and One’s Ideal Body: Lesbian versus Heterosexual Women

The measures of central tendency for all BIMTM items can be found in Table 2. With regard to their actual body, no differences were found between lesbian and heterosexual women, either for muscularity, *U* = 34,641.50, *z* = − 0.26, *p* = .796,* r* = − .01, or for body fat, *U* = 32,996.50, *z* = − 1.20, *p* = .230,* r* = − .05. With respect to their ideal body, no differences were found for muscularity, *U* = 33,595.00, *z* = − .85, *p* = .396,* r* = − .04, but lesbian women chose a silhouette that had significantly more body fat than did heterosexual women, *U* = 31,446.00, *z* = − 2.33, *p* = .020,* r* = − .10. However, after controlling for alpha inflation, the latter difference failed to reach statistical significance.

**Table 2:** Means (M), standard deviations (SD), and medians (Mdn) of the Body Image Matrix of Thinness and Muscularity variables

BIMTM Variables	1. Gay (*n* = 192)		2. Lesbian (*n* = 201)		3. Heterosexual female (*n* = 349)		4. Heterosexual male (*n* = 151)		Group comparisons and effect sizes Mann–Whitney *U* tests (Cohen’s *r*)
	*M (SD)*	*Mdn*	*M (SD)*	*Mdn*	*M (SD)*	*Mdn*	*M (SD)*	*Mdn*	
Actual body (M)	2.59 (1.69)	2.00	2.20 (1.60)	2.00	2.13 (1.50)	2.00	2.87 (1.67)	3.00	1 versus 4: *n.s.,* 2 versus 3: *n.s.*
Actual body (BF)	4.66 (1.52)	5.00	4.33 (1.36)	4.00	4.16 (1.23)	4.00	4.15 (1.57)	4.00	1 versus 4: *n.s.,* 2 versus 3: *n.s.*
Ideal body (M)	3.99 (1.66)	4.00	2.67 (1.68)	2.00	2.52 (1.59)	2.00	4.13 (1.77)	4.00	1 versus 4: *n.s.,* 2 versus 3: *n.s.*
Ideal body (BF)	3.01 (1.40)	3.00	3.30 (0.80)	3.00	3.18 (0.78)	3.00	3.04 (1.29)	3.00	1 versus 4: *n.s.,* 2 versus 3: *n.s*
Ideal partner (M)	4.02 (1.79)	4.00	2.34 (1.59)	2.00	3.22 (1.46)	3.00	2.25 (1.26)	2.00	1 > 3* (*r* = -.22), 2 versus 4: *n.s.*
Ideal partner (BF)	3.18 (1.63)	3.00	3.75 (0.92)	4.00	3.42 (1.21)	4.00	3.53 (0.87)	3.00	1 versus 3: *n.s.,* 2 versus 4: *n.s.*
Expected Body (M)	3.59 (1.61)	4.00	2.05 (1.31)	2.00	2.14 (1.45)	2.00	3.67 (1.73)	4.00	1 versus 4: *n.s.,* 2 versus 3: *n.s.*
Expected Body (BF)	3.11 (1.53)	3.00	3.59 (0.93)	3.00	3.40 (0.89)	3.00	3.34 (1.45)	3.00	1 versus 4: *n.s.,* 2 versus 3: *n.s.*

BIMTM: Body Image Matrix of Thinness and Muscularity; M: muscularity; BF: body fat; *n.s.* = not significant

*p < .001 (alpha adjustment led to a lowered significance threshold of *p* = .0031)

#### Comparison of One’s Actual and One’s Ideal Body: Gay versus Heterosexual Men

With regard to their actual body, gay and heterosexual men did not differ in perceived muscularity, *U* = 12,846.00, *z* = − 1.86, *p* = .063,* r* = − .10, but gay men perceived themselves as fatter (i.e., more body fat) than did heterosexual men, *U* = 12,188.50, *z* = − 2.59, *p* = .010,* r* = − .14. This difference disappeared after controlling for alpha inflation. Regarding the ideal body, no differences were found for muscularity, *U* = 13,806.50 *z* = − 0.77, *p* = 0.442,* r* = − .04, or for body fat, *U* = 14,129.00, *z* = − .41, *p* = 0.680,* r* = − .02.

#### Ideal Body of a Potential Romantic Partner: Lesbian Women versus Heterosexual Men

The two groups did not differ with respect to the preferred muscularity of a potential partner, *U* = 13,945.50 *z* = − 1.37, *p* = 0.172,* r* = − .07, but lesbians preferred a female body with more body fat than did heterosexual men, *U* = 13,037.50 *z* = − 2.44, *p* = .015,* r* = − .13. However, this difference failed to reach statistical significance after alpha adjustment.

#### Ideal Body of a Potential Romantic Partner: Gay Men versus Heterosexual Women

Compared to heterosexual women, gay men preferred a potential male partner who was more muscular, *U* = 24,897.00 *z* = − 5.04, *p* < .001,* r* = − .22, and thinner (i.e., less body fat), *U* = 28,929.50 *z* = − 2.73, *p* = 006,* r* = − .12. However, after alpha adjustment, only the former effect remained statistically significant.

#### Body Expected by a Potential Romantic Partner: Lesbian versus Heterosexual Women

With respect to muscularity, no differences were found between lesbian and heterosexual women regarding the body expected by a potential romantic partner, *U* = 34,376.00 *z* = − .41, *p* = .679,* r* = − .02. However, heterosexual women chose an expected body that was thinner (i.e., less body fat), *U* = 31,374.50 *z* = − 2.24, *p* = .025,* r* = − .10, though this effect fell prey to alpha adjustment.

#### Body Expected by a Potential Romantic Partner: Gay versus Heterosexual Men

Gay and heterosexual men did not differ in the silhouettes they chose for the body they believed would be expected by a potential romantic partner, either in terms of muscularity, *U* = 14,384.00 *z* = − .13, *p* = .901,* r* = − .01, or in terms of body fat, *U* = 13,055.00 *z* = − 1.61, *p* = .107,* r* = − .09.

#### Comparison of One’s Own Ideal Body and the Ideal Body of a Potential Romantic Partner: Gay Men

No difference was found between the silhouettes gay men chose for their own ideal body and the ideal body of a potential romantic partner, either in terms of muscularity, *z* = -0.05, *p* = 0.957, *r* = -0.03, or in terms of body fat, *z* = -1.80, *p* = 0.072, *r* = -0.09.

#### Comparison of One’s Own Ideal Body and the Ideal Body of a Potential Partner: Lesbian Women

Lesbian women chose a silhouette for the ideal body of a potential romantic partner that was significantly less muscular, *z* = − 4.48, *p* < 0.001, *r* = − .22, and had more body fat, *z* = − 6.47, *p* < 0.001, *r* = − .32, compared to the silhouette they chose for their own ideal body.

### Correlation Analysis

The correlation data can be found in Table 3. For all four subgroups, a higher estimation of one’s actual body fat on the BIMTM item actual body was associated with a higher degree of drive for thinness and eating pathology. For gay men, more muscular partner ideals were related to higher scores on each questionnaire. Especially for heterosexual women and men, the muscularity dimension of the BIMTM item expected body was positively correlated with each questionnaire score. Small associations of the BIMTM variables with age were found for all subgroups: Lower estimations of one’s actual and ideal body fat were related to a lower age in all participants. For women, a lower age was also associated with lower body fat estimations with respect to the ideal partner (i.e., lesbian and heterosexual women) and the expected body by a potential romantic partner (i.e., lesbian). Only for gay men were more muscular partner ideals related to a lower age. Associations between the BIMTM variables and educational level were only found for gay men: Higher estimations of one’s actual and ideal body fat were associated with a lower educational level.

**Table 3:** Spearman correlations between the Body Image Matrix of Thinness and Muscularity and the body image variables, eating pathology, age, and education for the four subgroups

BIMTM variables	Gay					Lesbian					Heterosexual female					Heterosexual male				
	DT^a^	DMS^b^	EDE-Q^c^	Age	EY	DT^a^	DMS^b^	EDE-Q^c^	Age	EY	DT^a^	DMS^b^	EDE-Q^c^	Age	EY	DT^a^	DMS^b^	EDE-Q^c^	Age	EY
AB (M)	.11	.02	.03	.04	.08	.15*	.17*	.13	.07	.01	.13*	.10	.12*	− .04	− .02	.12	.11	.18	.03	.02
AB (BF)	.43**	− .24**	.35**	.23**	− .17*	.47**	− .06	.51**	.22**	− .04	.29**	.07	.36**	.17**	− .09	.43**	− .17*	.29**	.23**	− .03
IB (M)	.05	.22*	.06	.05	− .03	.10	.46**	.09	.07	.05	.04	.27**	.06	.01	.06	.27**	.37**	.28**	− .10	.04
IB (BF)	− .01	− .27**	− .12	.14*	− .16*	.06	− .19**	.13	.27**	.03	− .07	− .02	− .01	.16**	− .04	− .02	− .32**	− .19	.16*	.07
IP (M)	.19*	.24**	.26**	− .16*	− .05	.04	.29**	.01	.10	.10	.05	.24**	.05	− .05	.09	.03	.05	.05	.05	− .10
IP (BF)	− .07	− .19*	− .14	.13	− .05	.08	− .08	.22**	.15*	− .05	− .06	− .17**	− .02	.16**	− .08	.15	− .21*	.04	.08	.11
EB (M)	.13	.20**	.22**	− .03	− .00	.10	.27**	.09	.05	.01	.15**	.21**	.20**	− .03	− .00	.26**	.34**	.44**	− .04	− .14
EB (BF)	.07	− .30**	− .16	.12	− .03	.16*	− .08	.11	.26*	.00	− .05	− .08	− .02	.10	− .05	.03	− .32**	− .12	.16	.13

BIMTM: Body Image Matrix of Thinness and Muscularity; AB: Actual body; IB: Ideal body; IP: Ideal partner; EB: Expected body; M: muscularity; BF: body fat; DT: Eating Disorder Inventory-2, subscale Drive for Thinness; DMS: Drive for Muscularity Scale; EDE-Q: Eating Disorder Examination-Questionnaire; EY: Education in Years

*p < .05; **p < .01

^a^*N* = 823: gay: *n* = 168; lesbian: *n* = 188; heterosexual female *n* = 327; heterosexual male *n* = 140

^b^*N* = 801: gay: *n* = 164; lesbian: *n* = 184; heterosexual female *n* = 317; heterosexual male *n* = 136

^c^*N* = 667: gay: *n* = 128; lesbian: *n* = 150; heterosexual female *n* = 279; heterosexual male *n* = 110

## Discussion

The main aim of the present online study was to investigate differences in appearance-related partner preferences between homosexual and heterosexual women and men and their associations with body image and eating pathology. The partner preferences were examined from two complementary perspectives, incorporating one’s own appearance-related ideals of the body of a potential romantic partner and the expectations of one’s own appearance in order to attract a potential romantic partner. Both aspects of partner preferences were measured using a newly designed silhouette measure that differentiates between the body fat and muscularity dimension. Based on previous research, it was assumed that appearance-related partner preferences would be more crucial for heterosexual women and homosexual men, as both groups try to attract male romantic partners, and men appear to place more importance on physical appearance than do women (e.g., Legenbauer et al., 2009).

With regard to the self-reported body image and eating pathology, the present findings revealed a rather gender-specific pattern, with women displaying a higher drive for thinness and men scoring higher on drive for muscularity. This is in line with the existing literature on general gender differences in body image (e.g., Karazsia et al., 2017; Kelley et al., 2010). In the same vein, both female groups reported more eating pathology than did heterosexual men, but did not differ among each other (see Yean et al., 2013) or from gay men. In accordance with previous findings (e.g., Peplau et al., 2009), gay men in the present sample reported a higher drive for thinness than did heterosexual men, and thus appeared to be slightly more vulnerable to a negative body image, whereas a lesbian orientation did not appear to have a protective effect on body image, as homosexual women did not differ from heterosexual women.

In support of the first hypothesis, no differences in perceptual body image in terms of the estimation of one’s own actual and ideal body were found between gay and heterosexual men, which mirrors the findings of Fussner and Smith (2015). However, contrary to our expectation, lesbian women’s own ideal body was not more corpulent (i.e., with more body fat) compared to that of heterosexual women. Even though this non-significant finding was due to alpha correction, the size of the effect was extremely small and therefore negligible. However, the finding does fit with data from qualitative research revealing that lesbian women also experience considerable sociocultural pressure to meet the thin body ideal (Smith et al., 2019). One reason for this may be found in the observable trend of increased acceptance of homosexuality in Western societies and media (e.g., Twenge et al., 2015), which may in turn also lead to increased acceptance of heteronormative beauty ideals within sexual minorities (Ahmad, & Bhugra, 2010; Smith et al., 2019).

The correlation analysis also revealed that only higher estimations of one's actual body fat, and not lower estimations of one’s actual muscularity, were consistently associated with at least increased drive for thinness and eating pathology across all groups. As there were no differences in BMI within the two female and the two male groups, this finding indicates that, irrespective of sex and sexual orientation, feeling fat seems to be more negative for one’s body image than feeling not muscular enough (see Cordes et al., 2016). This seems to be especially true for younger participants of the present study, as the correlation analysis showed that across all subgroups, a younger age was related to lower body fat estimations with respect to one’s ideal body.

Regarding the hypotheses concerning the differences in the appearance-related partner preferences between homosexual and heterosexual women and men, there was little evidence to support our assumptions. In accordance with our hypothesis, gay men preferred a more muscular build in their potential male partners compared to heterosexual women in their potential male partners, which underpins the relevance of physical attractiveness for homosexual men’s partner choices (see Legenbauer et al., 2009; Murnen et al., 2015). In line with this, the correlation data revealed that only for the group of gay men was preference for a more muscular build in a partner associated with increased drive for thinness, drive for muscularity, and eating pathology. Whether high demands on the physical appearance of a potential romantic partner negatively influence one's own body image, or whether the opposite is the case, cannot be answered by the present findings. However, it seems quite conceivable that gay men who place high demands on the muscularity of a potential romantic partner may be attracted to gay men who have similar demands on their partner’s appearance. This could open up a vicious circle of mutually reinforcing partner pressure that may lead to increased internalization of the mesomorphic body ideal (e.g., Tylka & Andorka, 2012) as well as to increased self-objectification (e.g., Engeln-Maddox et al., 2011).

However, contrary to our expectations, gay and heterosexual men did not differ in terms of the body they believe they should have in order to attract potential romantic partners. This may reflect the increasing relevance of physical appearance and body image for men in general (e.g., Fiske et al., 2014): particularly in times when the representation of one's own body and the social comparisons with others via social media is becoming increasingly ubiquitous (e.g., Fardouly & Vartanian, 2016). Moreover, the present finding also fits with findings by Pope et al. (2000), who reported that men tend to overestimate the degree of muscularity that women supposedly find attractive. This may also explain why, in the present study, heterosexual men’s expectations of how they should look in order to attract a potential romantic partner resemble the expectations of gay men, even though women place less importance on the physical appearance of their partners than do men (Legenbauer et al., 2009).

Furthermore, also contrary to our expectations, lesbian women did not choose silhouettes of a potential female romantic partner with more body fat compared to the silhouettes chosen by heterosexual men. Moreover, lesbian women displayed expectations regarding how they should look in order to attract a potential romantic partner that were comparable to the expectations of heterosexual women. However, these non-significant results were due to alpha correction. Again, the present findings may reflect the above-mentioned increased adoption of heteronormative standards of beauty within the lesbian community (Smith et al., 2019). Beyond this, the present findings are also in line with a qualitative study in lesbian and bisexual women by Huxley et al. (2011), who concluded that same-sex relationships did not serve as a protective factor for women’s body image per se, but have the potential to do so if past experiences of appearance pressure in relationships were more positive than negative. However, with respect to correlation data in the present study, it is striking that the total number of significant correlations between the four partner preferences items and the measures of body image and eating pathology was the smallest in lesbian women compared to all other groups, which may indicate a lower relevance of appearance-related partner preferences for lesbian women’s body image.

This assumption is further supported by the current finding, in line with our hypothesis, that lesbian women reported higher body-related beauty ideals with regard to body fat and muscularity for themselves than for a potential romantic partner, which could be interpreted as some sort of appearance-related double standard (Voges et al., 2019). This double standard may indicate that lesbian women: while adopting socio-cultural or heteronormative beauty ideals for themselves: are less likely to apply those ideals to their partners, at least compared to homosexual men, which may culminate in less body image-related partner pressure (see Huxley et al., 2015). In contrast, gay men apply the same beauty ideal standards to themselves as they do to a potential romantic partner.

Several limitations of the present study should be mentioned. First, the groups differed slightly in age, with homosexual men being older than both female groups, and heterosexual men being older than heterosexual women. As the literature shows that body image in men varies across different age groups, with younger men reporting a greater desire for a lean and muscular body than older men (e.g., McNeil & Firman, 2014), it is conceivable that the differences found in the present study between homosexual men and heterosexual women regarding the ideal body of a potential romantic partner might have been even more pronounced if the gay men in the present study had been somewhat younger. This assumption is further supported by the correlation analysis, which revealed that only for gay men was age negatively correlated with the muscularity dimension of the BIMTM variable partner ideal: Younger gay men preferred a more muscular partner. Second, in the same vein, the groups also differed in their educational level, with homosexual participants reporting fewer years of education compared to heterosexual participants. Again, only for gay men was a lower educational level associated with higher body fat estimations of one’s actual and ideal body. Thus, it is possible that the lower educational level in gay men may have affected the comparison with heterosexual men. Third, especially in comparison to lesbian women, heterosexual women in the present sample were more likely to be in a committed relationship. Although the investigation of partner preferences referred to a potential romantic partner, it cannot be completely ruled out that the results of the present study were affected by the mental representation of one's own current partner, whose physical appearance does not necessarily have to match the physical partner ideal. However, a restriction to non-partnered participants would have lowered the generalizability of the study, which is already limited as the present findings were obtained from a non-representative sample. Fourth, the sample size of the present study was too small to detect possibly existing small differences. Therefore, it is quite conceivable that some of the results that fell prey to alpha adjustment would have remained if the sample size had been larger. Finally, future research in this field may also benefit from investigating partner preferences in women and men with a bisexual orientation in order to test whether appearance-related partner preferences vary as a function of one’s own gender and the gender of a female and male partner, respectively.

### Conclusion

The present study adds to the very limited body of evidence on appearance-related partner preferences, and is the first study to our knowledge to investigate two complementary aspects of appearance-related partner preferences (i.e., one’s own partner ideal and one’s own expectations about how one should look in order to attract a potential romantic partner) and their associations with body image and eating pathology in homosexual and heterosexual women and men. Taken together, we found that gay men place higher demands on the muscular appearance of their potential romantic partners than do heterosexual women (see Legenbauer et al., 2009; Murnen et al., 2015), which may evoke increased partner pressure to fulfill the sociocultural beauty ideal. Furthermore, higher demands on the muscularity of a potential romantic partner were associated with increased drive for thinness and muscularity as well as with increased eating pathology in gay men. However, homosexual women did not show different partner preferences compared to heterosexual women and men, and a lesbian orientation did not appear to be protective for body image, contrary to some previous literature (e.g., Alvy, 2013).

As the differences found for gay men were small, the present data rather suggest that homosexual and heterosexual men and women share relatively comparable standards of beauty in terms of their own appearance and the appearance of a potential romantic partner. This may be indicative of the increased hegemony of heteronormative beauty ideals in Western societies for women (i.e., thin) and for men (i.e., lean and muscular) in general (see Ahmad, & Bhugra, 2010; Smith et al., 2019). Although the present study had no clinical focus, the correlation data at least emphasize that, irrespective of sexual orientation, body image-related prevention programs should target the possible negative effects of a pronounced critical self-view, especially in terms of the body fat dimension, as well as the possible negative effects of unrealistically high demands on the physical appearance of one’s own body and the body of a romantic partner on the body image.
